# An Onsite Calibration Method for MEMS-IMU in Building Mapping Fields

**DOI:** 10.3390/s19194150

**Published:** 2019-09-25

**Authors:** Sen Li, Yunchen Niu, Chunyong Feng, Haiqiang Liu, Dan Zhang, Hengjie Qin

**Affiliations:** 1 School of Building Environment Engineering, Zhengzhou University of Light Industry, 5 Dongfeng Road, Zhengzhou 450002, China; lisen@zzuli.edu.cn (S.L.); niuyunchen@zzuli.edu.cn (Y.N.); fcyong@zzuli.edu.cn (C.F.); zhangdan@zzuli.edu.cn (D.Z.); 2 China Academy of Electronics and Information Technology, 11 Shuangyuan Road, Beijing 100041, China; lhq0617@mail.ustc.edu.cn; 3 XinJiang Lianhai INA-INT Information Technology Ltd., 567 Dongrong Street, Urumqi 830000, Xinjiang, China

**Keywords:** building mapping, LiDAR, MEMS-IMU, error calibration, robot, BIM

## Abstract

Light detection and ranging (LiDAR) is one of the popular technologies to acquire critical information for building information modelling. To allow an automatic acquirement of building information, the first and most important step of LiDAR technology is to accurately determine the important gesture information that micro electromechanical (MEMS) based inertial measurement unit (IMU) sensors can provide from the moving robot. However, during the practical building mapping, serious errors may happen due to the inappropriate installation of a MEMS-IMU. Through this study, we analyzed the different systematic errors, such as biases, scale errors, and axial installation deviation, that happened during the building mapping, based on a robot equipped with MEMS-IMU. Based on this, an error calibration model was developed. The problems of the deviation between the calibrated and horizontal planes were solved by a new sampling method. For this method, the calibrated plane was rotated twice; the gravity acceleration of the six sides of the MEMS-IMU was also calibrated by the practical values, and the whole calibration process was completed after solving developed model based on the least-squares method. Finally, the building mapping was then calibrated based on the error calibration model, and also the Gmapping algorithm. It was indicated from the experiments that the proposed model is useful for the error calibration, which can increase the prediction accuracy of yaw by 1–2° based on MEMS-IMU; the mapping results are more accurate when compared to the previous methods. The research outcomes can provide a practical basis for the construction of the building information modelling model.

## 1. Introduction

Building information modelling (BIM) is rapidly growing and expanding. It has provided a critical solution to overcome the issues raised by the traditional methods [1,2]. BIM has been adopted by many new buildings, where a large number of old buildings also require the construction of a BIM model to benefit their operation and maintenance that can be consistent and available to the current technologies. A large portion of the construction of the BIM model still largely relies on Autodesk Computer Aided Design(AutoCAD) drawings, which show low efficiency, high consumption, and a long process time. These problems become even obvious when the target is a big and complex building. Therefore, light detection and ranging (LiDAR) has been largely adopted to acquire critical information for BIM models [3,4].

**Figure 1:**
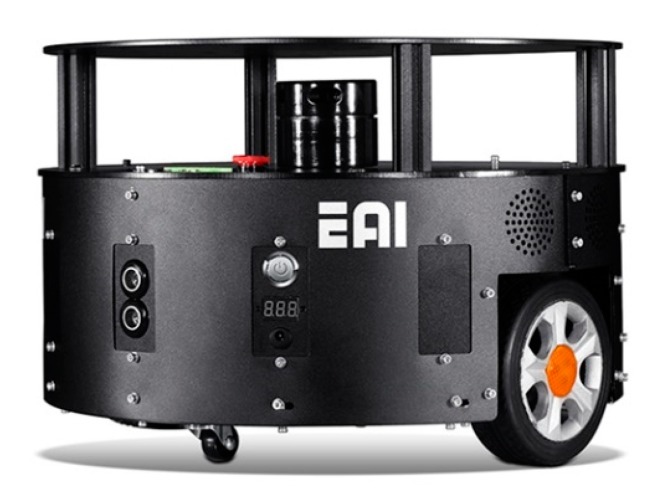
Laser simultaneous localization and mapping (SLAM) algorithm robot.

Simultaneous localization and mapping (SLAM) coupling the synchronous positioning based on light detection and ranging (LiDAR) is one of the most frequently adopted technologies for building mapping [5]. A reference robot is shown in Figure 1. SLAM is a mapping technology based on the combined synchronous positioning and the information of the environment. The positioning sensors mainly include a LiDAR sensor, an odometer, and an inertial measurement unit (IMU). The LiDAR is utilized to detect the surrounding environment, the odometer the moving distance, and IMU provides the related information of yaw. The gesture of the robot is then obtained by combining the information of the IMU and odometer. The measurement accuracy is an important influencing factor for obtaining the gesture of the robot, determining the accuracy of the building mapping [6].

To improve the mapping accuracy, Smith and Cheeseman [7] proposed an extended Kalman filter SLAM (EKF-SLAM) in 1986, which adopted the maximum likelihood estimation for the related data transformation. However, EKF-SLAM needs to calculate a large number of characteristic points, showing weak robustness. The obtained map is constructed by those characteristic points that are not good for the obstacle avoidance of the robot. Later, Montemerlo et al. [8] developed the Fast SLAM based on the Rao-Blackwellized particle filters. This algorithm considers the motion of the robot across time as a moving particle, obtained the weights of those particles based on the developed observation equation, and then constructs the occupancy grid mapping based on the gesture information. Compared to the feature map, the occupancy grid mapping favors routine planning for the robot more. However, Fast SLAM still showed two disadvantages: (a) as each particle contains the gesture information of the robot at the surrounding environment, the requirement of the computing resource is much higher, as calculations get heavier in a big space with a large number of particles; (b) the diversity of the particles decreases due to the increased resampling of those particles, such that the mapping accuracy is seriously affected.

Under the rapid development of construction technologies, the buildings are getting bigger and more complicated. The increase in resampling is a big issue for the related building mapping, and many studies have been undertaken to solve the resultant problems. For example, Grisetti et al. [9] developed a Gmapping algorithm. During the measurement, the difference between the predicted and real gesture is checked to determine if resampling is needed. The resampling is not necessary if the difference is small enough. The algorithm can largely reduce the number of calculations and the requirement of the computing resources. Based on those advantages, G mapping became one of the most popular methods for SLAM, but it still shows shortcomings, especially in large spaces, over long measurement times, and when dealing with accumulated errors. The current IMU with high prediction accuracy is expensive while its big volume limits its wide adoption in building mapping. Therefore, to solve the problem, it is critical to improve the measurement accuracy of that IMU, which could then largely reduce the calculation tasks. That is especially important for the ease of measurement when using the aforementioned small robots.

**Figure 2:**
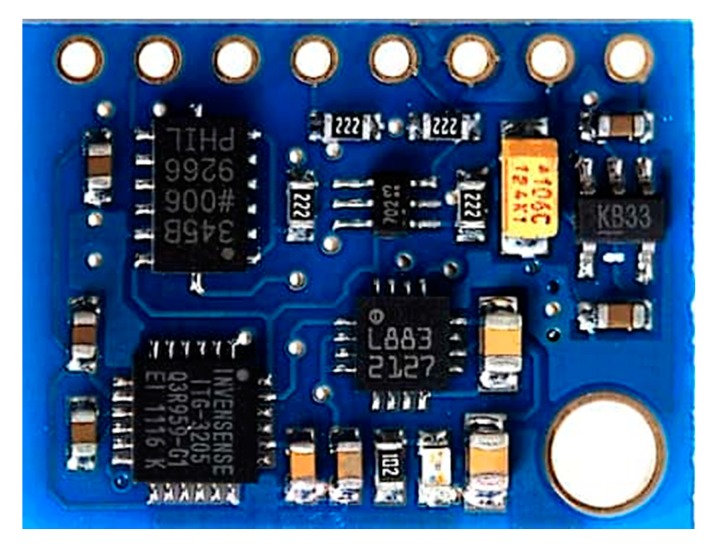
Micro electromechanical-inertial measurement unit (MEMS-IMU) product map.

Although the adoption of a highly-accurate IMU can improve the measurement accuracy of yaw, it is usually used in large equipment, not for the mapping of buildings. For example, Qureshi et al. [10] adopted the Xsens Mti-G710 at the high price of $2000. However, with the development of information technology, new low-cost and small-sized sensors have enabled many innovative methods in the field of building surveying to be achieved, which provides a new level of accuracy for the researchers in the BIM field [11]. For example, a typical micro electromechanical (MEMS)-IMU, including an accelerometer and a gyroscope, as seen in Figure 2, is only about $10–$50. It shows several advantages, such as a small volume, being lightweight, a low cost, impact resistance, and high reliability. It has been frequently adopted in building mapping [12,13,14]. However, these advantages are also accompanied with the reduction of measurement accuracy by errors. According to the sources, the errors can be further divided into systematic errors and random noise [15]. The random noise is a type of external noise during the data transformation of the MEMS-IMU, which reflect its random changing process. The coefficient for the random noise can be obtained by plotting Allan standard deviation and regression [16,17]. The random noise normally takes about 10% of the overall errors, which is the reason why it is usually ignored during practical applications [18]. The systematic errors are mainly because of the biases, scale errors, and installation axial deviation. Biases is related to a discrete model for the random walk, which can be considered as Brownian motion. The biases are different each time after switching on the machine as they are dependent on the many factors, such as internal structure and the temperature of the sensors [19].

Scale errors happen during the transformation of those digital signals, such as those from angular velocity and acceleration after the measurement. The axial installation deviation is because of the differences between the coordinates of the MEMS-IMU system and the basic one for the robot. Many studies have been undertaken regarding that systematic aspect. The traditional calibration method is based on the maximum likelihood estimation of the temperature compensation and then the calibrations of positions and velocities based on a three-axis turntable. The method is not viable for the onsite calibration, as the whole calibration process usually needs about four days, based on its use in big equipment [20,21]. The most common system error calibration for IMU is the IFBA method, which is based on an inertial coordinate. It uses the Earth’s rotation rate and gravity to calibrate the IMU error. However, for the low-cost MEMS-IMU, the measurement noise is much larger than the Earth’s rotation rate in the IFBA method, which causes the practicability of the IFBA method in MEMS-IMU error calibration to become very weak. Consider this: Haifeng Xing et al. [22] introduced RMT (rotary modulation technology) into the IFBA method, and applied a STF (strong tracking filter) algorithm to suppress the random noise of MEMS-IMU, which improved the calibration accuracy of MEMS-IMU greatly. However, that method still needs the support of a rotating platform, and is not suitable for the onsite calibration. Syed et al. [23] coupled GPS and IMU systems and compared it with the on-site calibration, where it cannot be applied to indoor calibration under the weak GPS signal indoors. Hwangbo et al. [24] proposed a self-calibration method based on factorization. Xiao et al. [25] developed an online IMU self-calibration method for a visual-inertial system equipped with a low-cost inertial sensor. These two studies adopted the calibration method with the assistance of the camera. Gravity acceleration is an effective reference during IMU calibration because the gravity acceleration does not change with the IMU rotation [26]. Tedaldi et al. [27] proposed a new onsite calibration method without the need for big equipment. The reasons for systematic errors were addressed by assuming that gravity’s acceleration and temperature keep constant. It detects the static interval by adopting a parameter-less static filter for the calibration. However, the method ignored the scenario when the calibrated plane is not perpendicular to gravity’s acceleration.

According to the above literature review, the current calibration methods based on MEMS-IMU are still bearing with the following shortcomings: (a) they are largely relying on the external calibration equipment that is much more expensive than the adopted MEMS-IMU itself; (b) the adopted GPS system is not viable for the indoor calibration due to the weak signal; and (c) they do not consider the issue that MEMS-IMU is not parallel to the horizon. To overcome the above-mentioned shortcomings, this study proposed an error calibration model to allow an easy and highly-accurate calibration based on MEME-IMU, without the need for external equipment. The research outcomes provide a basis for low-cost on-site indoor map building.

## 2. Development of Error Calibration Model

**Figure 3:**
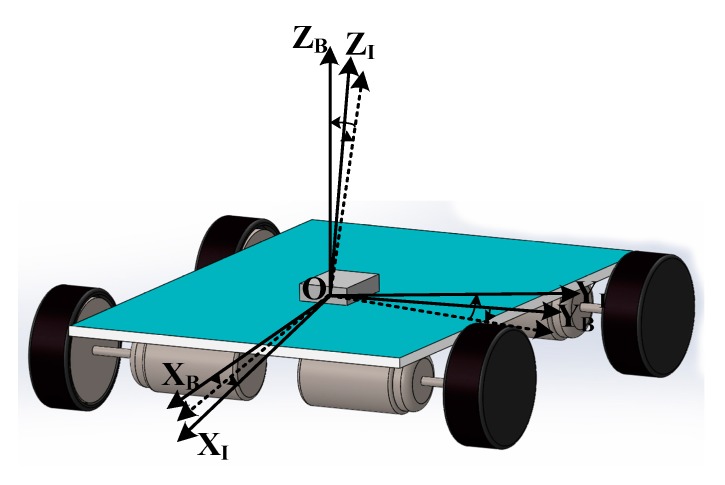
IMU and mobile car installation model.

The MEMS-IMU system was directly fixed to a movable car such that its movement was the same as the car’s, forming a strap-down inertial navigation system, as shown in Figure 3. Ideally, the coordinates of both MEMS-IMU and the car should be the same. However, during the practical applications, the chips of accelerometer and gyroscope were directly welded onto the printed circuit board (PCB) of the MEMS-IMU system, and then put into the system in the movable robot. During the whole process, the axial installation deviation happened due to the horizontal differences between the PCB and chips, and the coupling differences along the vertical direction between the MEMS-IMU system and the robot. A large number of studies indicated that the axial installation deviation would not change too much with changes of either time or temperature [28]. In this study, we focused on the calibration of the systematic errors due to the biases, scale errors, and installation axial deviation. The theoretical models and calibration principles for the accelerometer and gyroscope for the strap-down inertial measurement system are similar to the above calibration. Therefore, in the following contents, we focused on one type.

**Figure 4:**
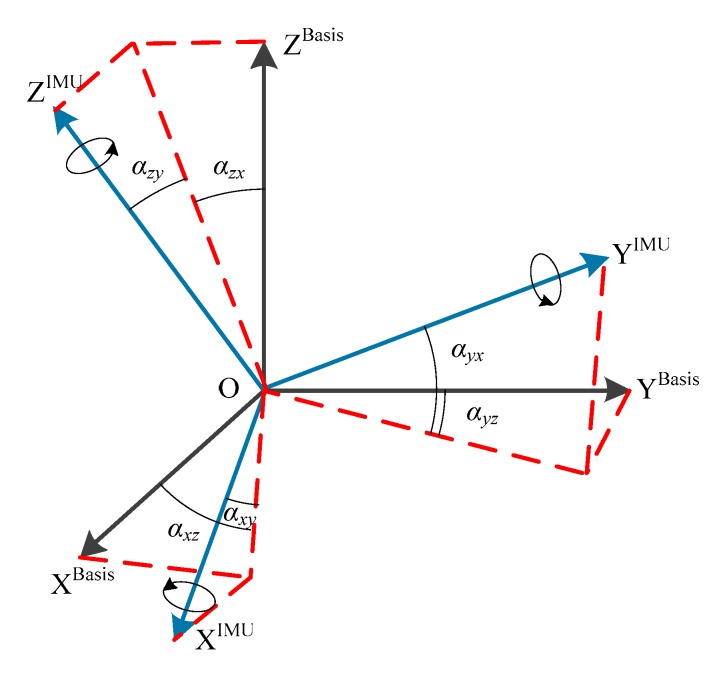
Schematic diagram of shaft declination.

As shown in Figure 3, we built a Cartesian coordinate based on the origin point of the center of the car, represented by B(x, y, z). Based on the same origin point, we also built a coordinate for the IMU system, denoted as I(x, y, z). Ideally, I(x, y, z) should be a Cartesian coordinate. However, it was not a perfect Cartesian coordinate due to manufacturing errors. During the measurement, the acquirement of the travelling distance of the car is based on B, whereas the others, such as LiDAR and odometer, used I. Therefore, there needed to be a process of converting the data in I to B. The converting process can be seen in Figure 4.

[X_i,_ Y_i,_ Z_i_] represent the non-Cartesian coordinates, whereas [X_b_, Y_b_, Z_b_] are the Cartesian coordinates. OX’ is the projection of OX_i_ on the plane of O-X_b_Z_b_. The same rules apply to OY’ and OZ’. The projection of IMU measurement data on the three axes can be seen in Table 1.

The *a_b_* is the IMU data in coordinate B, so the IMU coordinate and the coordinate of the car can be related through

(1) $$\left[\begin{array}{c} a_{bx}\\ a_{by}\\ a_{bz} \end{array}\right]=\left[\begin{array}{ccccccc} \cos\alpha_{xz}\cos\alpha_{xy} & & & -\cos\alpha_{yx}\cos\alpha_{yz} & & & \sin\alpha_{zy}\\ \sin\alpha_{xz} & & & \cos\alpha_{yx}\cos\alpha_{yz} & & & -\cos\alpha_{zy}\cos\alpha_{zx}\\ -\cos\alpha_{xz}\cos\alpha_{xy} & & & \cos\alpha_{yx} & & & \cos\alpha_{zy}\cos\alpha_{zx} \end{array}\right]\left[\begin{array}{c} a_{ix}\\ a_{iy}\\ a_{iz} \end{array}\right]$$

In practice, α is a very small angle. Therefore, we can assume that sinα = α and cosα = 1. The above equation can be then rewritten as,

(2) $$\left[\begin{array}{c} a_{bx}\\ a_{by}\\ a_{bz} \end{array}\right]=\left[\begin{array}{ccccccc} 1 & & & -\alpha_{yz} & & & \alpha_{zy}\\ \alpha_{xz} & & & 1 & & & -\alpha_{zx}\\ -\alpha_{xy} & & & \alpha_{yx} & & & 1 \end{array}\right]\left[\begin{array}{c} a_{ix}\\ a_{iy}\\ a_{iz} \end{array}\right],\;T=\left[\begin{array}{ccccccc} 1 & & & -\alpha_{yz} & & & \alpha_{zy}\\ \alpha_{xz} & & & 1 & & & -\alpha_{zx}\\ -\alpha_{xy} & & & \alpha_{yx} & & & 1 \end{array}\right]$$

For the calibration of IMU date, besides the rotation-matrix information during the installation, the scale errors and biases are also needed to be considered, which can be expressed by,

(3) $$\left\{ \begin{array}{l} a_{t}^{x}={Scale}^{x}\times (a_{m}^{x}+v^{x})\\ a_{t}^{y}={Scale}^{y}\times (a_{m}^{y}+v^{y})\\ a_{t}^{z}={Scale}^{z}\times (a_{m}^{z}+v^{z}) \end{array}\right.$$

where *a_t_* are the real data; *a_m_* are the MEMS-IMU measured data; *Scale*s are the scale errors; *v*s are the biases.

The above equation can be then changed to,

(4) $$\left[\begin{array}{c} a_{t}^{x}\\ a_{t}^{y}\\ a_{t}^{z} \end{array}\right]=\left[\begin{array}{ccccccc} {Scale}^{x} & & & 0 & & & 0\\ 0 & & & {Scale}^{x} & & & 0\\ 0 & & & 0 & & & {Scale}^{x} \end{array}\right]*\left(\left[\begin{array}{c} a_{m}^{x}\\ a_{m}^{y}\\ a_{m}^{z} \end{array}\right]+\left[\begin{array}{c} v^{x}\\ v^{y}\\ v^{z} \end{array}\right]\right)$$

After combining with the axial deviation, scale errors and biases, the error model of MEMS-IMU can be given by,

(5) $$\left[\begin{array}{c} a_{t}^{x}\\ a_{t}^{y}\\ a_{t}^{z} \end{array}\right]=\left[\begin{array}{ccccccc} 1 & & & -\alpha_{yz} & & & \alpha_{zy}\\ \alpha_{xz} & & & 1 & & & -\alpha_{zx}\\ -\alpha_{xy} & & & \alpha_{yx} & & & 1 \end{array}\right]*\left[\begin{array}{ccccccc} {Scale}^{x} & & & 0 & & & 0\\ 0 & & & {Scale}^{y} & & & 0\\ 0 & & & 0 & & & {Scale}^{z} \end{array}\right]*\left(\left[\begin{array}{c} a_{m}^{x}\\ a_{m}^{y}\\ a_{m}^{z} \end{array}\right]+\left[\begin{array}{c} v^{x}\\ v^{y}\\ v^{z} \end{array}\right]\right)$$

## 3. Error Calibration of MEMS-IMU System

In Equation (5), there are 12 unknowns,

$$\left(\begin{array}{cccccccccccccccccccccccccccccccccc} \alpha_{xz} & & & \alpha_{xy} & & & \alpha_{yz} & & & \alpha_{yx} & & & \alpha_{zy} & & & \alpha_{zx} & & & {Scale}^{x} & & & {Scale}^{y} & & & {Scale}^{z} & & & v^{x} & & & v^{y} & & & v^{z} \end{array}\right)$$

To solve the equation, Equation (5) can be changed to homogeneous equation,

(6) $$\left[\begin{array}{c} a_{t}^{x}\\ a_{t}^{y}\\ a_{t}^{z}\\ 1 \end{array}\right]=\left[\begin{array}{cccccccccc} {Scale}^{x} & & & -\alpha_{yz} & & & \alpha_{zy} & & & v^{x}\\ \alpha_{xz} & & & {Scale}^{y} & & & -\alpha_{zx} & & & v^{y}\\ -\alpha_{xy} & & & \alpha_{yx} & & & {Scale}^{z} & & & v^{z}\\ 0 & & & 0 & & & 0 & & & 1 \end{array}\right]\left[\begin{array}{c} a_{m}^{x}\\ a_{m}^{y}\\ a_{m}^{z}\\ 1 \end{array}\right]$$

After the transposition, it can be rewritten as,

(7) $$\left[\begin{array}{cccccccccc} a_{m}^{x} & & & a_{m}^{y} & & & a_{m}^{z} & & & 1 \end{array}\right]\left[\begin{array}{cccccccccc} {Scale}^{x} & & & \alpha_{xz} & & & -\alpha_{xy} & & & 0\\ -\alpha_{yz} & & & {Scale}^{y} & & & \alpha_{yx} & & & 0\\ \alpha_{zy} & & & -\alpha_{zx} & & & {Scale}^{z} & & & 0\\ v^{x} & & & v^{y} & & & v^{z} & & & 1 \end{array}\right]=\left[\begin{array}{cccccccccc} a_{t}^{x} & & & a_{t}^{y} & & & a_{t}^{z} & & & 1 \end{array}\right]$$

Based on the least-squares method, Equation (7) can be solved and the solution is,

*P* = (*X*^T^*X*)^−1^*X*^T^*Y*

where *P* can be rewritten as,

$$P=\left[\begin{array}{cccccccccc} {Scale}^{x} & & & \alpha_{xz} & & & -\alpha_{xy} & & & 0\\ -\alpha_{yz} & & & {Scale}^{y} & & & \alpha_{yx} & & & 0\\ \alpha_{zy} & & & -\alpha_{zx} & & & {Scale}^{z} & & & 0\\ v^{x} & & & v^{y} & & & v^{z} & & & 1 \end{array}\right]$$

In the above equations, *P* is the coefficient matrix to be solved, *X*s are the calibrated values for the three axes, denoted as [*a_t_^x^*, *a_t_^y^*, *a_t_^z^*]; and *Y* is the measured value for each of the three axes; namely [*a_m_^x^*, *a_m_^y^*, *a_m_^z^*].

The related data measurement is based on the calibration of the six sides, as shown in Table 1. During the calibration, those data were measured by MEMS-IMU when the six sides of the IMU (i.e., Z+, Z−, Y+, Y−, X+, and X−, as shown in Table 2) were facing upwards and kept still, where the related axis was parallel to the direction of gravity’s acceleration. The calibration of the accelerometer was based on the static data, while the calibration of gyroscope relied on those static data and also gravity’s acceleration.

**Figure 5:**
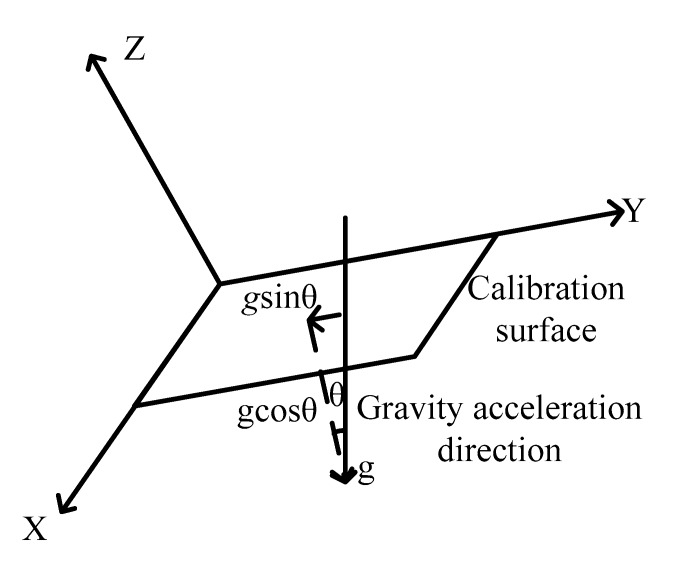
This is a schematic diagram of the deviation of the calibration surface from the direction of gravity’s acceleration.

During the six-sided calibration method, each rotation requires expensive rotating equipment to make sure the MEMS-IMU system is strictly parallel to the horizon to ensure only one side faces gravity’s acceleration at one time. However, the cost of the rotating equipment is even expensive than those MEMS-IMU systems with high accuracy. It is inevitable that the calibrated side is not perpendicular to the gravity’s acceleration, as shown in Figure 5. Taking step 1 for example, namely the upward Z-axis, the measured data should be (g, 0, 0) if the MEMS-IMU system is parallel to the horizon. As it is quite hard to achieve this, there should be an axis deviation angle. If we divide the measured gravity’s acceleration following different axes, the measured value can be expressed by [gcosθ, gsinθ, 0] ^T^. This problem should be solved to improve the prediction accuracy.

**Figure 6:**
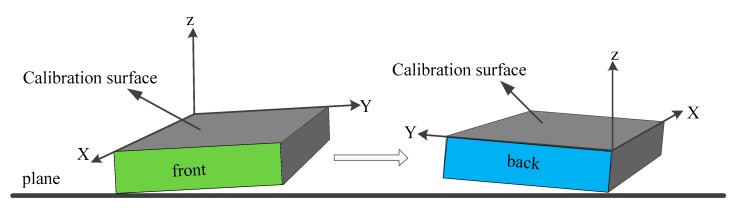
Schematic diagram of two rotations of the same calibration surface.

To avoid the situation, an improved six-sided calibration method was proposed through this study. The implementation processes can be summarized as,

- Start measuring the data after positioning the MEMS-IMU system on the floor; in order to avoid the singularity of the *P* matrix in the solution process, the data acquisition time should be extended to 3–5 s, to ensure the number of collected datums is greater than the unknown parameters in the equations.
- Rotate the calibrated side by 180° horizontally, as seen in Figure 6. Stay still for 3–5 s to measure the data;
- The finally measured data can be obtained by averaging the data obtained during the previous two steps;
- Repeat the calibration for the other five sides following the above three steps.

When comparing to the traditional method, our proposed calibration method adopts an extra horizontal rotation with two groups of data measurement to get the average. In a scenario where there is a deviation angle between the calibrated side and horizon, the measured data are [gcosθ, −gsinθ, 0] ^T^ and [gcos(π−θ), −gsin(π−θ), 0] ^T^, respectively. Usually, the deviation angle is quite small, these two groups of measured data can be then expressed by [g, −gθ, 0] ^T^ and [g, gθ, 0] ^T^, respectively. It can be seen that the deviation angle is gone after averaging these two groups of measured data; [g, 0, 0] ^T^. Therefore, the two measurements before and after the 180° horizontal rotation are useful to exclude the influences from the deviation angle. This is quite a reliable method for the onsite calibration without the need for the expensive equipment.

## 4. Experimental Validation

### 4.1. Hardware

**Figure 7:**
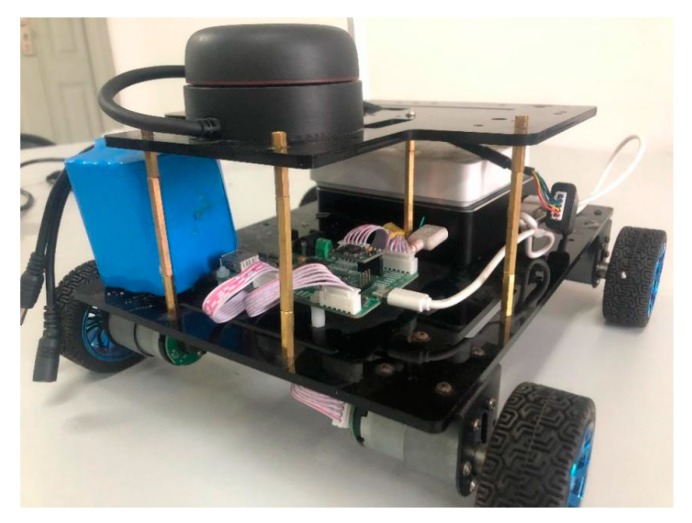
Rikirobot.

**Figure 8:**
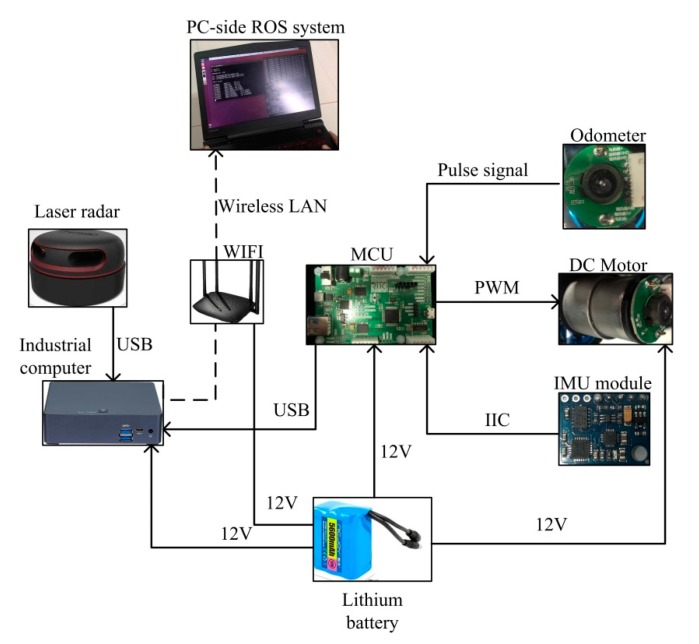
Overview of the hardware design.

The experiment was based on the rikirobot, as shown in Figure 7. We placed a MEMS-IMU system (GY-85, ADI, Norwood, MA, USA) on the rikirobot, coupled with a microcontroller unit (MCU, STM32F103, STMicroelectronics, Geneva, Switzerland) and a LiDAR (RPLIDAR-A2, SLAMTEC, Shanghai, China). The GY-85 MEMS-IMU system contains an accelerometer chip (ADXL345, ADI, MA, USA) and a gyroscope chip (ITG3205, ADI, Norwood, MA, USA), which communicate through the inter-integrated circuit (IIC) and MCU protocols. The overview of the hardware design can be seen in Figure 8.

### 4.2. The Software

The software for the experiment is programming based on a Robot Operating System (ROS) robot platform. This platform runs function modules individually for each node. These nodes are connected through an end-to-end topology when the platform is running. Each node contains a subprogram, where the related information exchange relies on the subscription and publishing functions. The related calibration program was developed based on the ROS software, which adopts the related gesture information for the map building.

**Figure 9:**
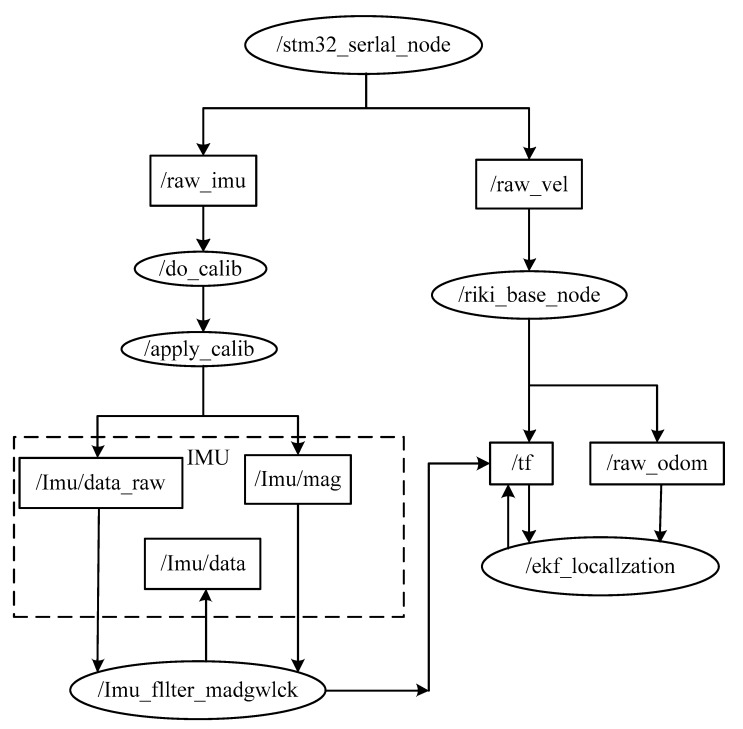
The flowchart of nodes in the software.

The flowchart of nodes in the software is shown in Figure 9. The data from the gyroscope are read from the chips, where the data are sent to the node/raw_imu. The error calibration node (do_calib) is used to collect the calibration data, and the node/apply_calib can solve the related equations and obtain the related solutions. After the subscription of those error calibration values, the IMU system then collects the data for yaw. All of these real time data are then subscribed by the robot control node (riki_base_node), where the transformation of coordinates is happening. The obtained yaw and odometer after the coordinate transformation are then used to confirm the gesture of the robot.

### 4.3. Experimental Results

The mean and variance of MEMS-IMU error parameters after ten groups of data collection can be seen in Table 3.

It can be seen from Table 3 that the errors for the Z-axis are relatively bigger than the other two axes. This is because of the suspension pin for the installation of the MEMS-IMU system on the robot, leading to the deviation in the center of gravity of the IMU system. On the other hand, the relatively bigger errors for the Z-axis are consistent with the installation, which proves that the related calibration model is viable. As the robot is moving on a plane, the relatively bigger errors along the Z-axis will not affect the collection of the related gesture information much, such as that of yaw.

The attitude angle information of the robot is collected by the gyroscope and accelerometer working together in the MEMS-IMU [29,30]. The gyroscope first measured the angular velocity, then, through an integral operation, the attitude angle value can be obtained. However, a measurement drift will appear in the gyroscope when it run a period time. In order to reduce the measurement error of the gyroscope, the accelerometer was used to measure the angle acceleration information, and another angular velocity was also can be calculated. Consider the stability of the accelerometer in a long time operation, the angular velocity which given by the accelerometer is taken as the state observation value, and the angular velocity in the gyroscope is deemed as the estimated value. Then, the state observation equation can be established (Equation (8)), and the final robot angle information will be acquired from solving the equation.

(8) Angle=∫(Gyro(k)+A(Gyro(k−1)−Accel(k−1)))dt

where *Gyro*(*k*) is the angular velocity value of the current moment measured by the gyroscope, *Gyro*(*k* − 1) is the value in the last moment, *A* is the gyroscope drift gain, and *Accel*(*k* − 1) is the angular velocity value obtained by the accelerometer.

To further validate the feasibility of the developed error calibration model, those error parameters were substituted into the model and then the related measurements were taken. As the robot was moving in a 2D environment on the building floor, we only measured the yaw during the experiments. The robot was rotated horizontally 30°, 45°, 60°, and 90°, respectively. Ten groups of data were measured and averaged for each rotation angle. The data before and after the calibration were compared, as shown in Table 4.

It can be seen from Table 4 that the average deviations of the yaw are about ±1° to ±3°, while after the calibration they are reduced to be within ±1°. Although an average deviation of ±3° for the yaw applies to those small maps, the application of the obtained map is limited because of the accuracy. For those big maps, it may lead to the failure of the building mapping. The calibration model developed in this study can control the deviation within ±1°, which is quite acceptable for the map building. Therefore, the developed calibration model can effectively improve the prediction accuracy of MEMS-IMU systems, so can largely reduce the computing requirements for the whole building mapping system.

## 5. Practical Implementation

**Figure 10:**
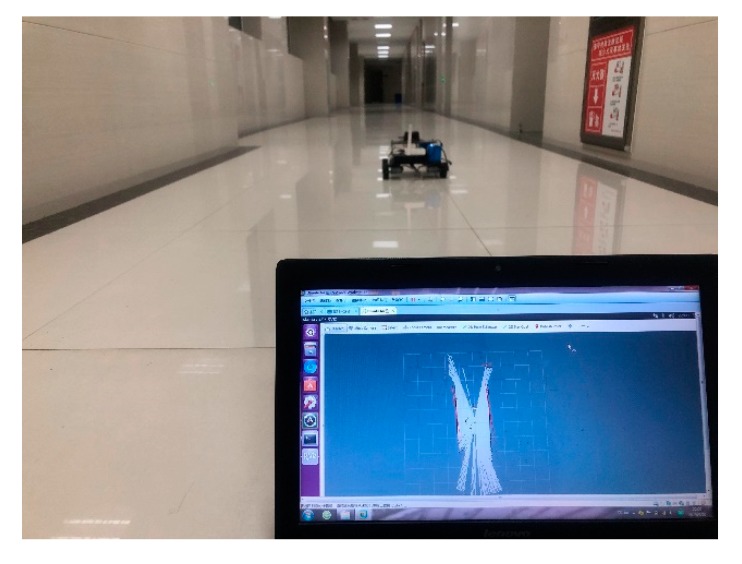
Map construction in a looped corridor.

**Figure 11:**
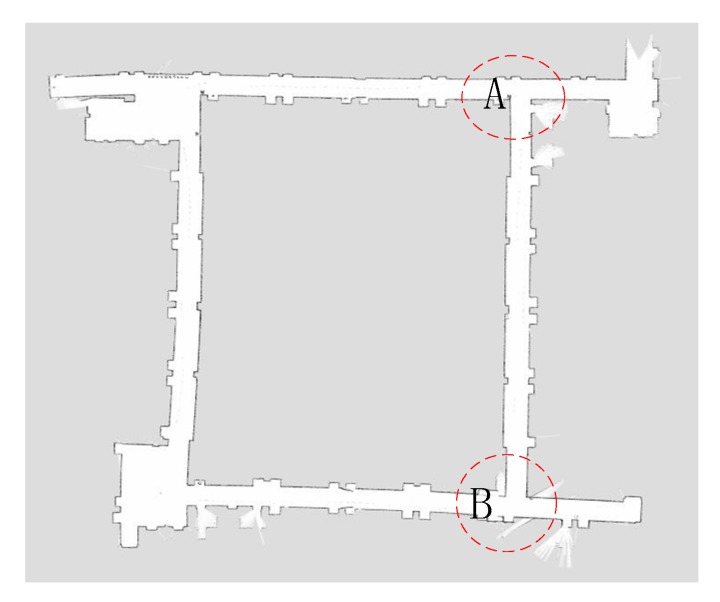
Map of the indoor looped corridor after the calibration.

To further validate the calibration model for the practical implementations, we selected a real circular corridor for application, as shown in Figure 10. The corridor is a loop; the width is 2.7 m and length is 130 m, which can be considered a big and complicated case, including many detailed structures, like the recessed doors and protruding pillars. The mapping process was based on the developed calibration model, where the map can be seen in Figure 11.

**Figure 12:**
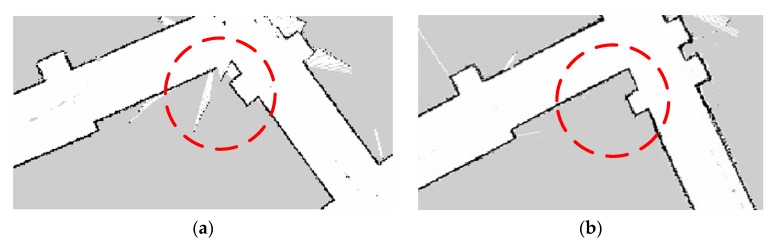
Map construction for turning right angle corridors before (**a**) and after (**b**) the calibration.

One of the challenging sections for a looped corridor is the corners, see Figure 12a,b. To show the advantage of the developed calibration model, we compared the map before and after the calibration, which is shown in Figure 12. At the corner A, the related yaw measurement had errors when the robot was moving to the corner. It resulted in an incorrect positioning of the robot that led to the deviation of the obtained map; the corner is not rectangular in the obtained map, as shown in Figure 12a. After adopting the calibration, it can be seen that the corner in the map becomes rectangular, which is consistent with reality. It was indicated that the calibration model is helpful to construct those detailed structures during the mapping.

**Figure 13:**
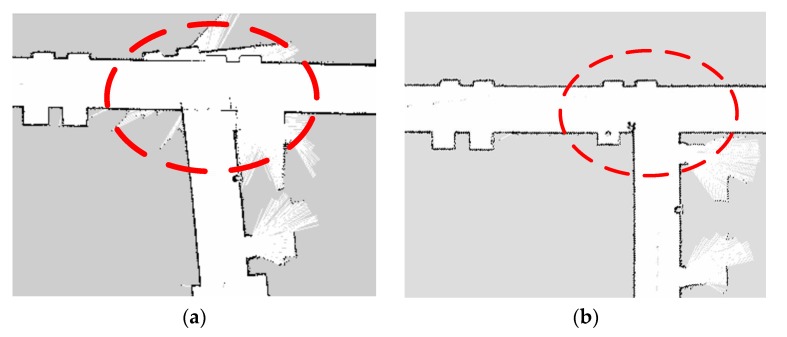
Ring corridor closed-loop connection for map construction before (**a**) and after (**b**) the calibration.

Corner B and the obtained map before and after the calibration can be seen in Figure 13a,b, respectively. It was known that the mapping time would be quite long due to the long looped corridor. The yaw was obtained by integrating the angular velocity of the gyroscope along with time, which is the reason for the continuing accumulation of errors. Under those circumstances, the data for the closed-loop detection was not accurate. The error calibration based on the MEMS-IMU can ensure accurate capturing of the gesture information by the robot when it is turning at the corner. That is beneficial to the construction of a closed-loop during the mapping.

Based on the above practical implementation, it is clear that the calibration model is useful for improving the accuracy of the building mapping and also enhancing the capture of those features of the buildings, such as a rectangular corner.

## 6. Conclusions

Based on a low-cost MEMS-IMU system, an error calibration method was proposed to overcome the shortcomings of traditional methods, such as relying on external equipment, expensive calibration auxiliary equipment and calibration errors. Through this study, we analyzed the different systematic errors, such as biases, scale errors, and axial installation deviation, which happened during the building mapping when using a robot equipped with a MEMS-IMU. It was indicated from the experiments that the proposed model is useful for the error calibration, which can increase the prediction accuracy of yaw by 1–2°. When based on the MEMS-IMU, the mapping results are more accurate compared to the previous methods. The research outcomes provide a basis for low-cost, on-site indoor map building.

## Figures and Tables

**Table 1:** Project of IMU measurement data on three axes.

X-Axis	Y-Axis	Z-Axis
*a_bxx_* = *a_ixx_*cos*α_xz_*cos*α_xy_*	*a_bxy_* = −*a_iy_*cos*α_yx_*sin*α_yz_*	*a_bxz_* = *a_iz_*sin*α_iz_*
*a_byx_* = *a_ix_*sin*α_xz_*	*a_byy_* = *a_iy_*cos*α_yx_*cos*α_yz_*	*a_byz_* = −*a_iz_*cos*α_zy_*cos*α_zx_*
*a_bzx_* = −*a_ix_*cos*α_xz_*sin*α_xy_*	*a_bzy_* = *a_iy_*cos*α_yz_*	*a_bzz_* = *a_iz_*cos*α_zy_*cos*α_zx_*

**Table 2:** Six-sided calibration method.

Z-Axis Upwards	Z-Axis Downwards	Y-Axis Upwards	Y-Axis Downwards	X-Axis Upwards	X-Axis Downwards
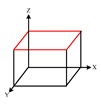	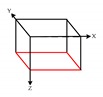	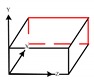	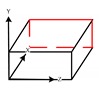	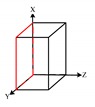	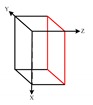

**Table 3:** Mean and variance of MEMS-IMU error parameters.

Parameter	Averaged Calibration Values (× 10^−2^)	Standard Deviation (× 10^−2^)
$\beta_{\mathrm{yz}}$	1.45	0.21
$\beta_{\mathrm{zy}}$	−5.59	0.01
$\beta_{\mathrm{xz}}$	−4.06	1.96
$\beta_{\mathrm{zx}}$	1.98	0.04
$\beta_{\mathrm{xy}}$	1.98	0.03
$\beta_{\mathrm{yx}}$	0.90	0.06
${Scale}^{x}$	98.52	0.10
${Scale}^{y}$	99.05	0.16
${Scale}^{z}$	99.17	0.11
$v^{x}$	0.37	0.05
$v^{y}$	2.54	0.53
$v^{z}$	57.25	4.21

**Table 4:** Experimental results of heading angle test.

Yaw	before Calibration	Error	after Calibration	Error
30°	27.6°	2.4°	29.6°	0.4°
45°	47.2°	−2.2°	45.9°	−0.9°
60°	61.8°	−1.8°	59.2°	0.8°
90°	88.0°	2.0°	90.5°	−0.5°
